# A parallel integrated learning technique of improved particle swarm optimization and BP neural network and its application

**DOI:** 10.1038/s41598-022-21463-2

**Published:** 2022-11-11

**Authors:** Jingming Li, Xu Dong, Sumei Ruan, Lei Shi

**Affiliations:** 1 School of Management Science and Engineering, Anhui University of Finance and Economics, Bengbu, 233030 Anhui China; 2 School of Finance, Anhui University of Finance and Economics, Bengbu, 233030 Anhui China

**Keywords:** Information technology, Computer science

## Abstract

Swarm intelligence algorithm has attracted a lot of interest since its development, which has been proven to be effective in many application areas. In this study, an enhanced integrated learning technique of improved particle swarm optimization and BPNN (Back Propagation Neural Network) is proposed. First, the theory of good point sets is used to create a particle swarm with a uniform initial spatial distribution. So a good point set adaptive particle swarm optimization (GPSAPSO) algorithm was created by using a multi-population co-evolution approach and introducing a function that dynamically changes the inertia weights with the number of iterations. Sixteen benchmark functions were used to confirm the efficacy of the algorithm. Secondly, a parallel integrated approach combining the GPSAPSO algorithm and the BPNN was developed and utilized to build a water quality prediction model. Finally, four sets of cross-sectional data of the Huai River in Bengbu, Anhui Province, China, were used as simulation data for experiments. The experimental results show that the GPSAPSO-BPNN algorithm has obvious advantages compared with TTPSO-BPNN, NSABC-BPNN, IGSO-BPNN and CRBA-BPNN algorithms, which improves the accuracy of water quality prediction results and provides a scientific basis for water quality monitoring and management.

## Introduction

With the advancement of current science and technology, optimization problems have developed rapidly and penetrated into a number of application areas, including financial investment^1^, engineering technology^2^, artificial intelligence^3^, pattern recognition^4^, and so on. However, the swarm intelligence algorithm has been extensively studied and developed, and it has been utilized to solve a number of optimization problems drawn from real-world issues with favorable outcomes, such as Genetic Algorithm (GA)^5^, Particle Swarm Optimization (PSO)^6^, Glowworm Swarm Optimization (GSO)^7^, Artificial Bee Colony (ABC)^8^, Invasive Weed Optimization (IWO)^9^, Cuckoo Search (CS)^10^, Bat Algorithm (BA)^11^, Fruit Fly Optimization (FOA)^12^, Whale optimization algorithm (WOA)^13^, Grey Wolf Algorithm (GWO)^14^, Ant Lion Optimization (ALO)^15^, Salp Swarm Algorithm (SSA)^16^, Butterfly Optimization Algorithm (BOA)^17^, Harris Hawk Optimization (HHO)^18^, Slime Mould Algorithm (SMA)^19^. Most of these swarm intelligence algorithms are generated by simulating the biological information system in the natural world. They have the characteristics of exploratory and development stages. To solve the optimization problem, they iteratively update existing solutions until they find the best one in the solution space. The main contributions of this paper are as follows.

- A new and improved adaptive particle swarm optimization algorithm based on good point set, adaptive inertia weight and multiple population co-evolution strategies is presented.
- A parallel integrated learning technique using the GPSAPSO algorithm and BPNN is proposed in order to create a water quality prediction model.
- By optimizing sixteen objective functions of various unimodal and multimodal types, the GPSAPSO algorithm's effectiveness has been measured.
- To evaluate water quality prediction model based on parallel integrated learning technique of GPSAPSO algorithm and BPNN, its performance has been compared with four well-known algorithms including TTPSO-BPNN, NSABC-BPNN, IGSO-BPNN and CRBA-BPNN.

The research in this paper mainly consists of the following contents: In the “Related works” section, BPNN, as one of the most commonly used machine learning algorithms, has many advantages and disadvantages. To address the shortcomings of BP neural network, it is proposed to optimize its network by using various swarm intelligence algorithms to improve the classification ability. Further, the particle swarm algorithm is introduced, and various improvement strategies of the particle swarm algorithm are presented to derive the research content of this paper; In the “Improved particle swarm algorithm” section, the traditional particle swarm algorithm is firstly introduced, and then an improved particle swarm algorithm based on good point set, adaptive inertia weight and multiple swarm co-evolution strategies is proposed; In the “Algorithm test” section, the performance of the improved particle swarm algorithm is tested by sixteen benchmark functions, and the effectiveness of the improved algorithm is further verified by comparing it with four swarm intelligence algorithms, namely, GPSAPSO, TTPSO, NSABC, IGSO and CRBA; In the “Algorithm application” section, the improved algorithm is used to optimize the BPNN and build a GPSAPSO-BPNN parallel integrated learning water quality prediction model for the Huai River; Finally, the “Conclusion” section summarizes the whole paper.

## Related works

Swarm intelligence algorithm serves as a useful model for further optimizing and enhancing the conventional machine learning algorithm. Machine learning, as the most widely used technology in the twenty-first century, has been favored by more and more scholars and has been employed in fields such as medical^20^, education^21^, industry^22^, finance^23^ and so on. For example, multilayer Long Short Term Memory (LSTM) networks are used for demand forecasting to address the high volatility of demand data^24^; Random Forest (RF) and Support Vector Machines (SVM) are also used for predictive mapping of aquatic ecosystems^25^; The dissolved oxygen in urban rivers is predicted and analyzed by Extreme Learning Machine (ELM) and Artificial Neural Network (ANN)^26^; BP Neural Network (BPNN) is used to establish the evaluation model of software enterprise risk^27^.

Among the machine learning algorithms, BPNN has become an important classification algorithm in the field of artificial intelligence because of its early development. It is also a widely used network model with strong nonlinear mapping ability, generalization ability and fault tolerance ability^28^. It is worth noting that many parameters of traditional machine learning algorithms have a large impact on performance. For example, BPNN is limited by initial weight and threshold size, resulting in its shortcomings of local optimal solution and poor prediction accuracy^29^. Therefore, many scholars have conducted a lot of research on the optimization and application of BP neural networks with the help of swarm intelligence algorithms. Grey Wolf algorithm (GWO)^30^, is used to optimize BPNN to predict short-term traffic flow; The improved fruit fly algorithm (FOA)^31^ is used to optimize the BPNN, and then predict the air quality and verify the superiority of the improved algorithm; The optimization algorithm ABC-BPNN is used to predict rock blasting and crushing^32^; GA-BPNN algorithm is used to test the materials^33^.

As a swarm intelligence algorithm proposed for a long time, the particle swarm algorithm has been applied and improved by many scholars during these years of development. An improved particle swarm algorithm based on the principle of clonal selection is proposed to force particles to jump out from stagnation by putting a hybrid mutation scheme, which in turn continues to search for optimal solutions^34^; Different location update strategies are used, and mutation operator is introduced to avoid falling into local optimal solution and enhance the ability of space development^35^; local best information is introduced in addition to personal and global best information, which in turn increases the diversity of the population^36^; Using the idea of system entropy (SR), a new iteration method of inertia weights and a local jump optimization strategy are introduced into the particle swarm optimization algorithm, and six high-dimensional functions are used to test the performance of the algorithm, and to verify the effectiveness of the optimization strategy^37^.

Considering that most of the improvements made by these scholars focus on the improvement of the particle swarm search process, which often greatly increases the complexity of the algorithm. Since the initial solutions of traditional particle swarm algorithm are not uniformly distributed in the solution space, it easily leads to the problems of instability and low computational accuracy of the algorithm. Then, the theory of good point set and variable inertia weight strategy are employed to improve the traditional particle swarm algorithm, and multi-population co-evolution strategy is also used to build a GPSAPSO algorithm based on good point and variable inertia weight. GPASPSO algorithm was tested with sixteen benchmark functions and compared with TTPSO^38^, NSABC^39^, IGSO^40^ and CRBA^41^ algorithms, the results show that GPSAPSO algorithm is superior to other algorithms in terms of optimal value, worst value, average value and variance.

Then, the improved GPSAPSO algorithm is used to optimize the parameters of BPNN, construct the parallel ensemble learning algorithm of GPSAPSO-BPNN, and apply it to the water quality prediction modeling of Huai River, so as to construct the Huai River water quality prediction model based on GPSAPSO-BP algorithm, and carry out the simulation experiment with the water quality sample data of Huai River from January to October 2021. The experimental results show that the classification accuracy of GPSAPSO-BPNN algorithm is greatly improved compared with traditional BPNN and other algorithms. Therefore, the GPSAPSO algorithm proposed in this paper is an effective and practical optimization algorithm.

## Improved particle swarm optimization algorithm

### Basic particle swarm optimization

The main idea of PSO algorithm is to regard each bird in the space as a particle of candidate solution, and find the optimal value of the individual by tracking the flight (motion) of the particle to find the optimal solution of the population, i.e., the information is shared and exchanged by each individual in the whole population so that the population position approaches the optimal position from the initial distribution position of the solution space and the optimal solution of the problem is obtained.

The core of the PSO algorithm is the speed and position update formula:

1 $$ v(d + 1) = wv(d) + c_{1} r_{1} (pbest(d) - x(d)) + c_{2} r_{2} (gbest(d) - x(d)) $$

2 $$ x(d + 1) = x(d) + v(d + 1) $$

In the formula, $d$ is the current iteration number, $pbest$ is the current optimal particle position, $w$ is the inertial weight, which is used to maintain the influence of particle speed. $gbest$ is the historical optimal particle location, $c_{1}$, $c_{2}$ are the individual and social learning factors, $v(d + 1)$ is the particle speed of the $d + 1$ iteration, $x(d)$ is the particle location of the $d$ iteration, $r_{1}$ and $r_{2}$ are the random numbers in (0, 1).

According to the above formula, the PSO algorithm records the historical optimal position of each particle and the historical optimal position of the whole particle population in the process of searching for the optimal solution, calculates the corresponding fitness value, compares the current fitness value with the historical optimal fitness value of the individual or population by continuously updating the particle position and velocity, and records the optimal value according to the objective function. Finally, the ideal individual positions and fitness values are generated.

### Algorithm improvement strategy

#### Good point set theory

The theory of good point set was proposed by Hua Luogeng and Wang Yuan^42^, its core ideas can be described as: Let $G_{d}$ be a unit cube in D-dimensional Euclidean space, if $r \in G_{d}$, then:

3 $$ P_{n} (k) = (\{ r_{1}^{(n)} \times k\} ,\{ r_{2}^{(n)} \times k\} ,...{ ,}\{ r_{d}^{(n)} \times k\} ) \, (k = 1,2,... \, ,n) $$

4 $$ \phi (n) = C(r,\varepsilon )n^{ - 1 + \varepsilon } $$

5 $$ r = \{ 2\cos (2\pi k/p),1 \le k \le d\} $$

$\{ r_{d}^{(n)} \times k\}$ in Eq. (3) takes the fractional part of {} and its error satisfies Eq. (4), where $C(r,\varepsilon )$ is a constant related only to r and $\varepsilon$, and is called Good Point Set (GPS). r is the good point, taken from Eq. (5). Where p satisfies the minimum prime number of $(p - 3)/2 \ge d$. A good point set is generated by exponential series method in this paper, that is, by $r = \{ e^{j} ,1 \le j \le d\}$, r is also a good point.

**Figure 1:**
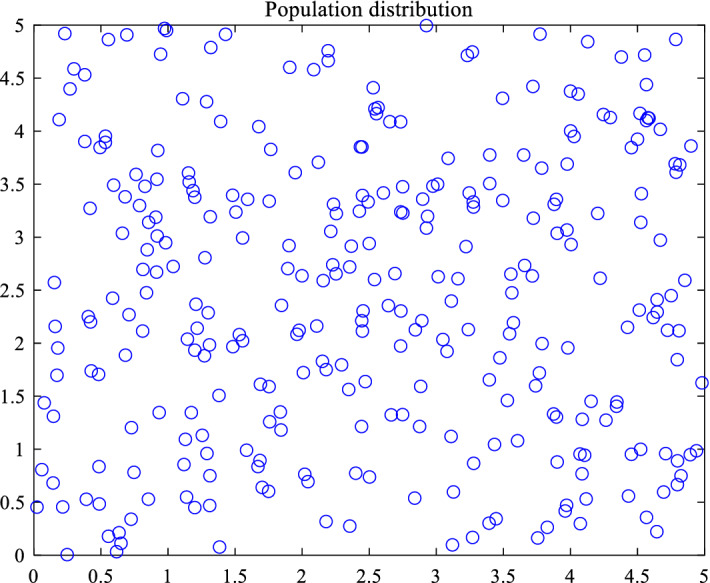
Random initialization.

**Figure 2:**
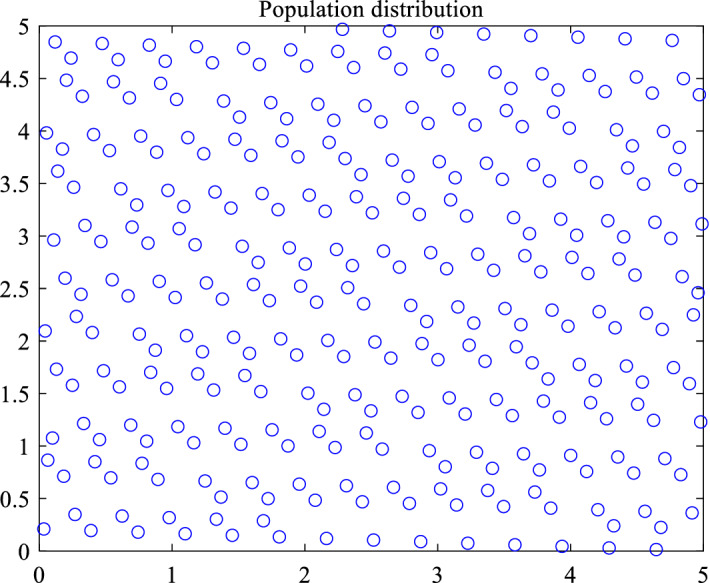
Good point set initialization.

Based on the above theory, a two-dimensional initial particle swarm distribution with a certain initial size is generated as shown below. Figure 1 is the initial population distribution generated by random mode, and Fig. 2 is the initial population distribution generated by good point set. It is obvious that the population generated by good point set is more uniform than that generated by random mode at the same initial population size, and the distribution of the initial population generated by good point set is determined in different experiments. It has been proved that it has sufficient stability. At the same time, in the solution of high-dimensional problems, the initial position is generated independently of the dimension, which ensures that it is more suitable for solving high-dimensional problems.

#### Adaptive inertia weight

The inertia weight in the PSO algorithm is a significant variable that reflects the system's ability to conduct both local and global searches. The larger inertia weight improves the global search ability, and the corresponding smaller inertia weight has strong local search ability, but it may fall into the local optimal solution. In the later stage, the smaller inertia weight is adopted to search the optimal value more accurately. The adaptive inertia weight strategy not only ensures the operation efficiency of the algorithm, but also improves the optimization ability of the algorithm. Then the speed update formula of GPSAPSO algorithm becomes Eq. (6), and the position update formula also changes with the speed update formula.

6 $$ v(d + 1) = w(d)*v(d) + c_{1} r_{1} (pbest(d) - x(d)) + c_{2} r_{2} (gbest(d) - x(d)) $$

7 $$ w(d) = ws - ((ws - we)/ger)*d $$

8 $$ w(d) = ws - (ws - we)*(d/ger)^{2} $$

**Figure 3:**
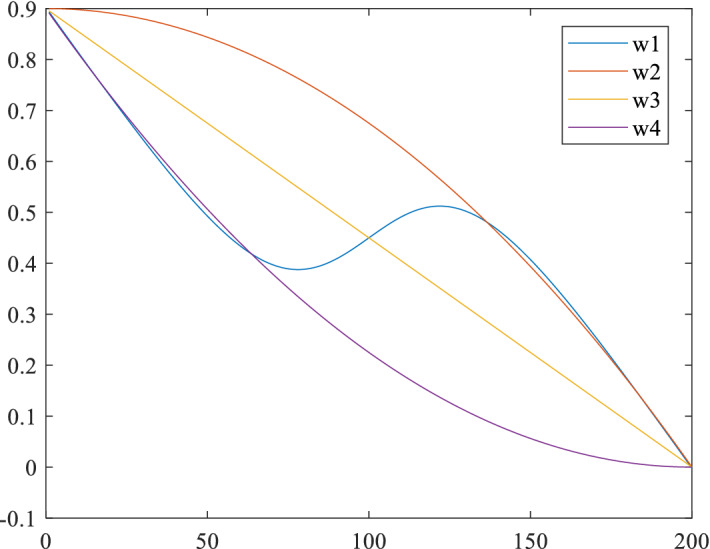
Inertia weight strategy.

At present, there are several commonly used decreasing inertia weights: linear decreasing strategy (W3), linear differential decreasing strategy (W2 and W4) and decreasing strategy with disturbance term (W1). The process of these methods following the number of iterations is shown in Fig. 3 Compared with the ordinary linear decline strategy, the inertia weight of the linear differential decline strategy follows the decline of the number of iterations more smoothly and more stable. The inertia weight commonly used in particle swarm optimization algorithm is 0.9. In order to make the later search strategy more accurate, we choose the decreasing interval of inertia weight as [0.9, 0.001].

#### Multi-population co-evolution strategy

**Figure 4:**
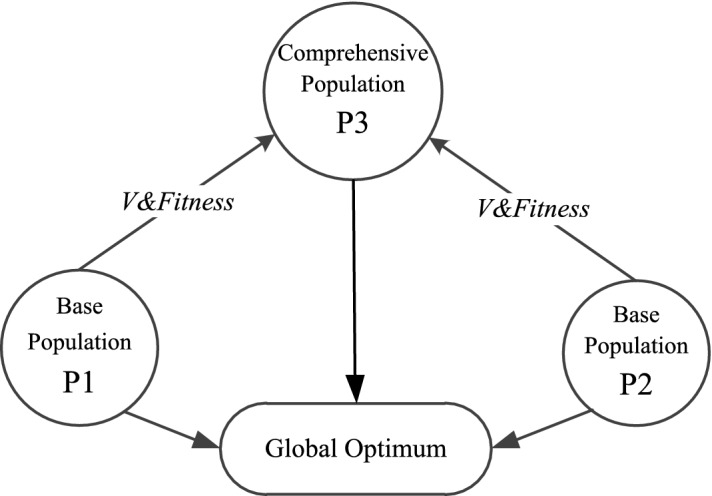
Multi-population strategy.

Numerous population co-evolutionary techniques are put forth in this research. The particle population is often divided into three subpopulations: (Base population), (Base population), and (Comprehensive Population). When looking for the optimum option in the solution space, these three subpopulations cooperate and communicate while updating iterations using various speed and location update formulae. In order to maintain the exchange and cooperation among the three populations, the speed update of ${P}_{3}$ in the integrated population depends on the speed and fitness value of ${P}_{1}$ and ${P}_{2}$ in the base population, and the location update also depends on the current and historical optimal location of the two base populations. The interaction and cooperation between these populations can be summarized in Fig. 4:

The $P_{1}$ and $P_{2}$ iteration equations for the base population are consistent with the above:

9 $$ v_{id}^{1(2)} (d + 1) = w(d) \cdot v_{id}^{1(2)} (d) + c_{1} r_{1} (pbest_{id}^{1(2)} (d) - x_{id}^{1(2)} (d)) + c_{2} r_{2} (gbest(d) - x_{id}^{1(2)} (d)) $$

10 $$ x_{id}^{1(2)} (d + 1) = x_{id}^{1(2)} (d) + v_{id}^{1(2)} (d + 1) $$

The iteration equation for the population $P_{3}$ is as follows:

11 $$ \begin{aligned}  v_{id}^{3} (d + 1) &= w(d) \cdot \left( {\frac{{m - m_{1} }}{m} \cdot v_{id}^{1} (d + 1) + \frac{{m - m_{2} }}{m} \cdot v_{id}^{2} (d + 1) + v_{id}^{3} (d)} \right)  \\ & \quad +c_{1} r_{1} (pbest_{id}^{3} (d) - x_{id}^{3} (d)) + c_{2} r_{2} (gbest(d) - x_{id}^{3} (d)) \\ \end{aligned} $$

12 $$ x_{id}^{3} (d + 1) = \alpha_{1} \cdot x_{id}^{3} (d) + \alpha_{2} \cdot pbest_{id}^{3} (d) + \alpha_{3} \cdot gbest(d) + v_{id}^{3} (d + 1) $$

where, the speed update formula of integrated population $P_{3}$ uses the base population $P_{1}$ and $P_{2}$ speeds, and the fitness values $m_{1}$ and $m_{2}$. $m$ is the sum of the current fitness of the two basic populations. Through Eq. (11), the basic population with better current fitness value has more influence on the integrated population. Equation (12) introduces the current optimal position $pbest_{id}^{3}$ and the historical optimal position $gbest$ on the basis of the original position update strategy. The reason is that the evolution mode of the particle swarm determines that the optimal position of the population plays a leading role in the flight of the particle swarm to the optimal solution. $\alpha_{1}$, $\alpha_{2}$ and $\alpha_{3}$ are set to 1/6, 1/3 and 1/2 respectively as the influence factors. The greater the value of $\alpha$, the greater the impact of this position on the comprehensive population position.

## The algorithm test

### Test functions

The effectiveness of the GPSAPSO algorithm will be examined in this section. To further highlight the performance of GPSAPSO, algorithm's effectiveness is tested by using sixteen benchmark functions and comparing it to TTPSO, NSABC, IGSO and CRBA algorithms. The last eight functions are multidimensional functions, while the first eight functions are two-dimensional functions. The specific function expressions are shown in Table 1. The sixteen functions in the experiment have minimal values, which are their ideal values.

**Table 1:** Test functions.

Function expression	Dim	Range
$f_{1} = x_{1}^{2} + 2x_{2}^{2} - 0.3 \times \cos \;(3\pi x_{1} ) \times \cos \;(4\pi x_{2} ) + 0.3$	2	$\left| x \right| \le 10$
$f_{2} = 100 \times (x_{1}^{2} - x_{2}^{2} ) + (1 - x_{1} )^{2}$	2	$\left| x \right| \le 10$
$f_{3} = 0.26 \times (x_{1}^{2} + x_{2}^{2} ) - 0.482x_{1} x_{2}$	2	$\left| x \right| \le 10$
$f_{4} = (x_{1} + 2x_{2} - 7)^{2} + (2x_{1} + x_{2} - 5)^{2}$	2	$\left| x \right| \le 10$
$f_{5} = (1.5 - x_{1} + x_{1} x_{2} )^{2} + (2.25 - x_{1} + x_{1} x_{2}^{2} )^{2} + (2.625 - x_{1} + x_{1} x_{2}^{3} )^{2}$	2	$\left| x \right| \le 10$
$f_{6} = x_{1}^{2} + x_{2}^{2} + 25(\sin^{2} x_{1} + \sin^{2} x_{2} )$	2	$\left| x \right| \le 2\pi$
$f_{7} = (\left| {x_{1} } \right| - 5)^{2} + (\left| {x_{2} } \right| - 5)^{2}$	2	$\left| x \right| \le 10$
$f_{8} = 0.5 + \frac{{\sin^{2} \sqrt {x_{1}^{2} + x_{2}^{2} } - 0.5}}{{[1.0 + 0.0001(x_{1}^{2} + x_{2}^{2} )]^{2} }}$	2	$\left| x \right| \le 10$
$f_{9} = \sum\limits_{i = 1}^{d} {(x_{1} )^{2} + 10\cos (2\pi x_{i} )^{2} + 10}$	20	$\left| x \right| \le 5.12$
$f_{10} = \sum\limits_{i = 1}^{d - 1} {100 \times (x_{i + 1} - x_{i}^{2} )^{2} + (1 - x_{i} )^{2} }$	20	$\left| x \right| \le 10$
$f_{11} = \sum\limits_{i = 1}^{d} {(x_{i}^{2} ) + \left( {\sum\limits_{i = 1}^{d} {0.5ix_{i} } } \right)^{2} + \left( {\sum\limits_{i = 1}^{d} {0.5ix_{i} } } \right)^{4} }$	20	$\left| x \right| \le 10$
$f_{12} = (1/4000)\sum\nolimits_{i = 1}^{d} {(x^{2} )} + \prod\nolimits_{i = 1}^{d} {\cos (x_{i} /i^{1/2} ) + 1}$	20	$\left| x \right| \le 60$
$f_{13} = \sum\limits_{i = 1}^{d} {(x_{i}^{2} )}$	20	$\left| x \right| \le 10$
$f_{14} (x) = - 20\exp \left( { - 0.2\sqrt {\frac{1}{n}\sum\limits_{i = 1}^{n} {x_{i}^{2} } } } \right) - \exp \left( {\frac{1}{n}\sum\limits_{i = 1}^{n} {\cos (2\pi x_{i} )} } \right) + 20 + e$	20	$\left| x \right| \le 32$
$f_{15} (x) = \sum\limits_{i = 1}^{n} {\left| {x_{i} } \right|} + \prod\limits_{i = 1}^{n} {\left| {x_{i} } \right|}$	20	$\left| x \right| \le 10$
$f_{16} (x) = \sum\limits_{i = 1}^{n} {\left( {\left| {x_{i} + 0.5} \right|} \right)^{2} }$	20	$\left| x \right| \le 10$

### Parameter setting

To better validate the performance of the GPSAPSO algorithm, improved algorithms of several traditional swarm intelligence algorithms^38–41^. Were used to compare with GPSAPSO. These improved algorithms have been applied and published in the 2021 IEEE Congress on Evolutionary Computation (CEC) conference as well as in some journals, and have been reasonably validated by the proposers. Comparing them with our proposed GPSAPSO algorithm can better prove the value of our improved version and highlight the effectiveness of the GPSAPSO algorithm.

Table 2 are the parameter setting contents of the several algorithms used. Since the GPSAPSO algorithm is an improved algorithm from the original algorithm PSO, the parameter settings are consistent with the original algorithm except for the improved part, so they are not given again. In order to ensure the reasonableness of the algorithm test, the initial population size $popsize$ is set to 100 for all algorithms, and the number of iterations $ger$ is set to 100 generations. The effectiveness of the improved GPSAPSO algorithm was verified by comparison experiments.

**Table 2:** Parameter setting.

Algorithm	Parameter content	Parameter setting
GPSAPSO	Inertia weight $ws, we$	$ws$ = 0.9, $we\in [$ 0.001,0.4]
	Self-learning factor ${c}_{1}$	${c}_{1}$ = 1.4995
	Social-learning factor ${c}_{2}$	${c}_{2}$ = 1.4995
TTPSO	Branching factor $d$	$d\ge 2$
	Topology	Tournament
	Inertia weight $w$	$w$ = 0.9
NSABC	Control parameter	$limit$ = 100
	Index of selected neighbor	$k$ = 10
	Parameter upper limit	$C$ = 1.5
IGSO	Initial fluorescein ${l}_{0}$	${l}_{0}$ = 5
	Initial step size $s$	$s$ = 0.5
	Fluorescein volatilization factor $\rho$	$\rho$ = 0.4
	Fluorescein renewal rate $\gamma$	$\gamma$ = 0.6
	Field change rate $\beta$	$\beta$ = 0.08
CRBA	Iteration parameter $\tau$	$\tau$ = 2.3
	Pulse loudness value ${R}_{0}$	${R}_{0}$ = 0.7
	Initial loudness ${A}_{0}$	${A}_{0}$ = 0.9

### Experimental result

The five algorithms, GPSAPSO, TTPSO, NSABC, IGSO and CRBA, are tested with sixteen benchmark functions, and 30 independent iterations are run for each algorithm in this section. The optimal value, the worst value, the mean and the variance of these sixteen functions are obtained as the main indicators of the superiority or inferiority of the two algorithms. At the same time, the convergence curves of the sixteen standard functions during the finite iterations are also recorded. The performance testing of the GPSAPSO algorithm is divided into two parts according to the types of low-dimensional and high-dimensional test functions. In addition to the parameter settings specified before the experiments, some parameters of the five algorithms need to be dynamically adjusted during the experiments according to the different test functions, so that the results of the test functions show the optimal effect. For example, the parameter *we* of the GPSAPSO algorithm has an important impact on the accuracy of the GPSAPSO algorithm for finding the best and the convergence speed, therefore, in order to balance the accuracy and convergence, a larger value of the parameter *we* between [0.001, 0.4] is chosen for the low-dimensional test function relative to the high-dimensional function.

Similarly, for the IGSO and GSO algorithms, the decision radius is also a parameter that has an important impact on the algorithm results. In the experimental process, according to the references and experimental experience, the appropriate decision radius is adjusted for different test functions, such as 2.448(${f}_{1}$), 2.548(${f}_{2}$), …, 30.048(${f}_{12}$), …, 32.048(${f}_{16}$). Finally, the five algorithms are tested in order of low-dimensional and high-dimensional test functions and obtained the final experimental results.

#### Low dimensional test function

**Figure 5:**
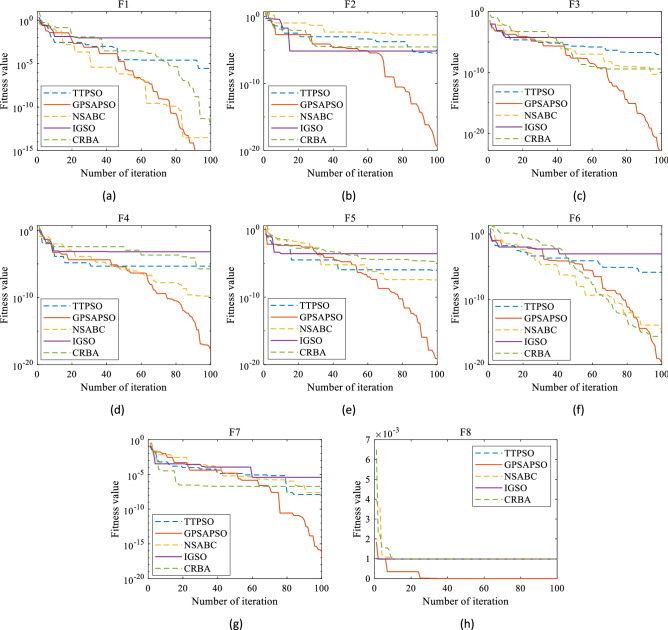
Iterative process of low dimensional function.

The results of low dimensional test functions are shown in Fig. 5 and Table 3. For low dimensional ${f}_{1}$ to ${f}_{3}$, ${f}_{5}$ to ${f}_{8}$, the average values obtained by running GPSAPSO algorithm 30 times are 3.70E − 18, 5.82E − 17, 1.43E − 19, 6.13E − 21, 1.92E − 24, 1.57E − 18 and 6.60E − 04 respectively, which are better than the average values obtained by TTPSO, NSABC, IGSO and CRBA, and the optimal value, worst value and variance are also better than the above algorithms, especially for ${f}_{2}$, ${f}_{5}$ and ${f}_{6}$, running 30 times results are much better than other algorithms. However, for ${f}_{4}$, the average running value of NSABC algorithm is 6.73E − 22, which is better than 3.37E − 21 of GPSAPSO algorithm. Through Fig. 5 and Table 3, it can be seen that GPSAPSO algorithm has great advantages in solution accuracy and stability compared with the other three algorithms.

**Table 3:** Results of 30 runs of low dimensional functions.

	TTPSO	NSABC	IGSO	CRBA	GPSAPSO
$${{\varvec{f}}}_{1}$$					
Worst	2.267721E − 05	2.553513E − 14	9.418810E − 02	8.178217E − 09	5.551115E − 17
Best	4.414684E − 08	1.110223E − 16	1.905193E − 05	1.156780E − 11	0
Mean	4.666975E − 06	2.967996E − 15	1.833889E − 02	1.384278E − 09	3.700743E − 18
Std	4.482296E − 11	2.677574E − 29	7.338375E − 04	3.271468E − 18	1.983486E − 34
Rank	4	2	5	3	1
$${{\varvec{f}}}_{2}$$					
Worst	1.637812E − 04	3.355315E − 04	1.086430E − 03	1.826331E − 06	4.739494E − 16
Best	4.531961E − 07	4.877187E − 08	1.086968E − 07	3.515622E − 10	6.102963E − 19
Mean	3.282511E − 05	1.535623E − 05	2.495193E − 04	4.888582E − 07	5.823536E − 17
Std	1.443323E − 09	3.713633E − 09	1.076127E − 07	3.217606E − 13	1.383771E − 32
Rank	4	3	5	2	1
$${{\varvec{f}}}_{3}$$					
Worst	6.997039E − 07	4.253731E − 09	1.336978E − 04	1.342945E − 09	2.699281E − 18
Best	1.069105E − 10	8.570840E − 12	9.207510E − 09	2.497696E − 12	9.769277E − 22
Mean	6.226152E − 08	6.990252E − 10	1.687869E − 05	1.852653E − 10	1.432911E − 19
Std	1.816296E − 14	6.807405E − 19	1.015946E − 09	7.165292E − 20	2.375116E − 37
Rank	4	3	5	2	1
$${{\varvec{f}}}_{4}$$					
Worst	7.080420E − 06	2.914983E − 21	2.358705E − 03	5.513886E − 07	3.123659E − 20
Best	2.044078E − 08	1.184143E − 23	6.723772E − 07	4.005156E − 10	4.410329E − 24
Mean	8.480835E − 07	6.733416E − 22	2.940743E − 04	7.065048E − 08	3.365373E − 21
Std	1.962630E − 12	5.699855E − 43	3.152841E − 07	1.111447E − 14	4.558172E − 41
Rank	4	1	5	3	2
$${{\varvec{f}}}_{5}$$					
Worst	2.072685E − 05	8.239942E − 08	2.776058E − 03	1.234201E − 07	6.379623E − 20
Best	2.887694E − 08	6.585241E − 11	1.626089E − 07	7.338608E − 12	4.779711E − 24
Mean	3.096056E − 06	1.491255E − 08	1.286446E − 04	2.189552E − 08	6.131498E − 21
Std	2.544625E − 11	2.800000E − 16	2.591209E − 07	8.247456E − 16	1.509203E − 40
Rank	4	2	5	3	1
$${{\varvec{f}}}_{6}$$					
Worst	5.778872E − 05	2.648856E − 14	5.862035E − 02	4.453918E − 17	3.500164E − 23
Best	1.170544E − 08	6.036982E − 18	4.322043E − 06	3.434451E − 20	3.447365E − 27
Mean	6.491699E − 06	2.026135E − 15	1.017376E − 02	8.332784E − 18	1.923361E − 24
Std	1.317390E − 10	6.155858E − 30	2.112588E − 04	1.046490E − 34	4.083310E − 47
Rank	4	2	5	3	1
$${{\varvec{f}}}_{7}$$					
Worst	7.264868E − 06	7.424328E − 08	3.368981E − 06	2.263041E − 08	1.268035E − 17
Best	1.526670E − 08	4.168165E − 13	1.539421E − 08	2.329014E − 11	2.869042E − 20
Mean	6.582430E − 07	5.756695E − 09	6.240637E − 07	4.049678E − 09	1.566034E − 18
Std	1.710319E − 12	2.118486E − 16	5.952302E − 13	2.573063E − 17	7.020742E − 36
Rank	5	3	4	2	1
$${{\varvec{f}}}_{8}$$					
Worst	9.855165E − 04	9.854326E − 04	9.865543E − 04	9.854327E − 04	9.854029E − 04
Best	2.463412E − 06	5.079149E − 07	9.854029E − 04	9.515640E − 04	1.665335E − 16
Mean	8.143831E − 04	8.599449E − 04	9.854420E − 04	9.842819E − 04	6.602198E − 04
Std	1.264072E − 07	1.062356E − 07	4.413118E − 14	3.818545E − 11	2.016311E − 07
Rank	2	3	5	4	1
Sum rank	31	19	39	22	9
Mean rank	3.875	2.375	4.875	2.75	1.125
Total rank	4	2	5	3	1

Generally speaking, in terms of low dimensional function, GPSAPSO algorithm is much better than TTPSO, IGSO and CRBA algorithm, and has obvious advantages in four aspects: optimal value, worst value, average value and variance. Compared with the NSABC algorithm, except that the GPSAPSO algorithm is inferior to the NSABC algorithm in ${f}_{4}$, the performance of the GPSAPSO algorithm is still superior in the other 7 low-dimensional test functions.

#### High dimensional test function

**Figure 6:**
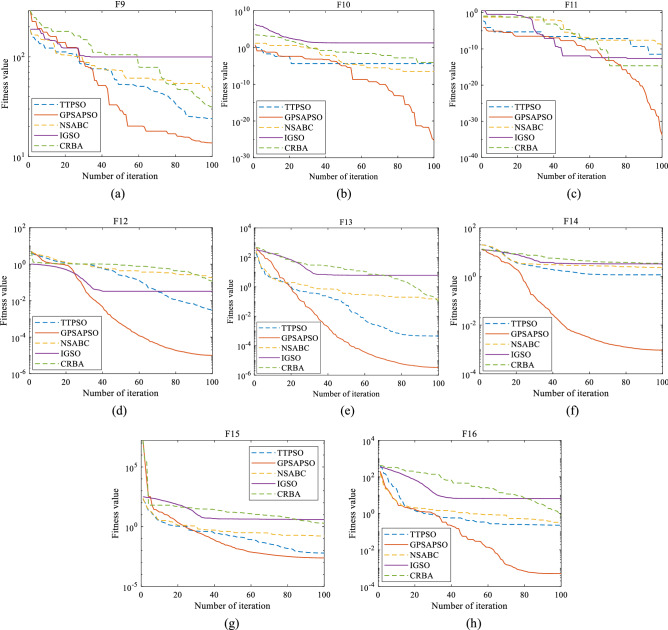
Iterative process of high-dimensional function.

The results of high-dimensional test functions are shown in Fig. 6 and Table 4. It can be seen that the performance of GPSAPSO algorithm on high-dimensional functions is significantly improved compared with TTPSO, NSABC, IGSO and CRBA algorithms. The average values of GPSAPSO algorithm running 30 times on high-dimensional test functions ${f}_{9}$ to ${f}_{16}$ are 1.71E − 01, 1.65E − 23, 1.19E − 28, 2.64E − 05, 3.12E − 06, 1.20E − 05, 4.21E − 03 and 3.90E − 03 respectively. For ${f}_{9}$, ${f}_{12}$ and ${f}_{16}$, the performance of GPSAPSO algorithm is better than that of TTPSO, NSABC, IGSO and CRBA algorithm, and the variance is smaller, which proves that it has better stability; For ${f}_{10}$, ${f}_{11}$, ${f}_{13}$, ${f}_{14}$ and ${f}_{15}$, the performance of GPSAPSO algorithm is significantly improved compared with other algorithms in several indicators.

**Table 4:** Results of 30 times of high-dimensional function operation.

	TTPSO	NSABC	IGSO	CRBA	GPSAPSO
$${{\varvec{f}}}_{9}$$					
Worst	6.289205E + 01	8.124867E + 01	9.702472E + 01	1.092325E + 02	3.282714E + 01
Best	1.494779E + 01	2.778917E + 01	8.563365E + 01	2.617890E + 01	9.248632E + 00
Mean	2.932179E + 01	4.825447E + 01	9.117730E + 01	6.658759E + 01	1.709459E + 01
Std	1.403382E + 02	1.614690E + 02	2.217917E + 01	4.616491E + 02	2.694716E + 01
Rank	2	3	5	4	1
$${{\varvec{f}}}_{10}$$					
Worst	2.815665E − 03	3.843360E − 04	2.249157E + 00	1.373550E − 04	1.974942E − 22
Best	2.112196E − 06	1.418602E − 07	7.441240E − 05	1.151703E − 07	4.142979E − 26
Mean	4.862784E − 05	5.370253E − 05	4.989150E − 01	2.463714E − 05	1.649918E − 23
Std	6.634009E − 08	7.924844E − 09	9.600330E − 01	8.167195E − 10	1.535172E − 45
Rank	3	4	5	2	1
$${{\varvec{f}}}_{11}$$					
Worst	1.147669E − 08	3.258578E − 07	8.826070E − 13	3.358709E − 10	1.802463E − 27
Best	0	5.086384E − 12	2.011935E − 18	1.530601E − 11	0
Mean	1.896004E − 09	4.428299E − 09	2.198771E − 14	2.496341E − 11	1.193353E − 28
Std	9.729818E − 18	8.582472E − 16	1.442906E − 25	4.444196E − 21	1.265816E − 55
Rank	4	5	2	3	1
$${{\varvec{f}}}_{12}$$					
Worst	4.907317E − 02	1.013322E + 00	3.851482E − 02	1.028376E + 00	1.252613E − 05
Best	9.577764E − 04	1.214192E − 02	1.507777E − 02	5.251707E − 01	5.579148E − 07
Mean	2.001901E − 02	5.164110E − 01	2.939266E − 02	8.635266E − 01	2.640530E − 05
Std	1.225827E − 04	2.061647E − 01	8.171326E − 05	2.107291E − 02	1.117796E − 08
Rank	2	4	3	5	1
$${{\varvec{f}}}_{13}$$					
Worst	4.280576E − 03	1.097016E + 00	5.685581E + 00	2.250238E + 00	1.317134E − 05
Best	5.556189E − 05	2.949492E − 01	4.321669E + 00	2.927199E − 02	3.171975E − 07
Mean	1.095807E − 03	6.564266E − 01	4.820608E + 00	3.321092E − 01	3.121793E − 06
Std	1.138349E − 06	5.705649E − 02	2.893478E − 01	1.879548E − 01	1.187090E − 11
Rank	2	4	5	3	1
$${{\varvec{f}}}_{14}$$					
Worst	3.225701E + 00	5.213369E + 00	3.701980E + 00	5.005182E + 00	2.288036E − 03
Best	1.158318E + 00	2.597105E + 00	3.161258E + 00	1.069870E + 00	4.914404E − 04
Mean	2.174902E + 00	3.837696E + 00	3.332169E + 00	2.336646E + 00	1.195734E − 03
Std	3.140219E − 01	4.970029E − 01	4.865447E − 02	8.365261E − 01	1.581415E − 07
Rank	2	5	4	3	1
$${{\varvec{f}}}_{15}$$					
Worst	5.023287E + 00	3.860776E + 00	1.165920E + 01	1.060255E + 01	2.036341E − 02
Best	1.731815E − 01	8.435587E − 01	7.927882E + 00	3.089337E − 01	3.702918E − 04
Mean	1.174665E + 00	1.936924E + 00	9.444504E + 00	1.603112E + 00	4.213966E − 03
Std	7.353417E − 01	7.053028E − 01	2.486917E + 00	4.061240E + 00	5.598226E − 05
Rank	2	4	5	3	1
$${{\varvec{f}}}_{16}$$					
Worst	9.431983E − 01	2.580797E + 00	6.463993E + 00	3.874423E + 00	9.578559E − 02
Best	3.790485E − 02	2.170260E − 01	2.769268E + 00	9.917564E − 02	2.480810E − 04
Mean	2.832213E − 01	7.399041E − 01	5.035652E + 00	6.792702E − 01	3.904931E − 03
Std	4.871374E − 02	1.801368E − 01	2.289568E + 00	7.346642E − 01	6.014328E − 03
Rank	2	4	5	3	1
Sum rank	19	33	34	26	8
Mean rank	2.375	4.125	4.25	3.25	1
Total rank	2	4	5	3	1

Figure 6 shows the iterative curves of GPSAPSO, TTPSO, NSABC, IGSO, and CRBA, and it is obvious that GPSAPSO has a large advantage over the other algorithms in terms of accuracy and convergence speed. Compared with the performance of low dimensional functions, it can be seen that GPSAPSO algorithm has greater advantages in solving high-dimensional function problems.

#### Statistical analysis

After testing the performance of the proposed GPSAPSO algorithm through sixteen low and high dimensional test functions, statistical analysis of the search results is required to ensure that the obtained results are statistically significant and to prove statistically the significance of the advantage of the GPSAPSO algorithm in the face of these selected competing algorithms.

The Wilcoxon rank sum test^43^ was used to implement the requirement for validating the effectiveness of the algorithm. The Wilcoxon rank sum test enabled the determination of the p-value between GPSAPSO and several other competing algorithms, and the improved algorithm was usually chosen to have a significant advantage over several competing algorithms when p < 0.05.

Tables 5 and 6 show the average ranking of the five algorithms and the simulation results of the Wilcoxon rank sum test, respectively. The smaller the average ranking in Table 5 proves that the algorithms perform better, and the results show that the GPSAPSO algorithm achieves the best performance compared with other algorithms in both low-dimensional and high-dimensional functions. The p-value between GPSAPSO and other algorithms is less than 0.05 for both low-dimensional and high-dimensional functions, which proves that GPSAPSO algorithm performs significantly better than TTPSO, NSABC, IGSO and CRBA.

**Table 5:** Mean ranking values of different algorithms.

Algorithms	Mean ranking	
	Low dimensional test function	High dimensional test function
TTPSO	3.875	2.375
NSABC	2.375	4.125
IGSO	4.875	4.25
CRBA	2.75	3.25
GPSAPSO	1.125	1

**Table 6:** Results of Wilcoxon rank sum test.

Compared algorithms	Function type	
	Low dimensional test function	High dimensional test function
GPSAPSO with TTPSO	6.9930E − 03	4.9883E − 02
GPSAPSO with NSABC	4.9883E − 02	1.4763E − 02
GPSAPSO with IGSO	2.9526E − 03	6.9930E − 03
GPSAPSO with CRBA	1.0412E − 02	2.0668E − 02

According to the above content, GPSAPSO algorithm should show higher stability than the basic particle swarm optimization algorithm, and the stable initial position uniformly generated through the good point set is more dominant in solving high-dimensional problems. These characteristics are reflected in the iterative process, optimal value and variance in Figs. 5, 6, Tables 3 and 4. At the same time, we also use Wilcoxon rank sum test to further prove the statistical advantages of GPSAPSO algorithm over several competitive algorithms. As a result, the GPSAPSO algorithm significantly outperforms the classic PSO algorithm in terms of performance.

## Algorithm application

### BP neural network

**Figure 7:**
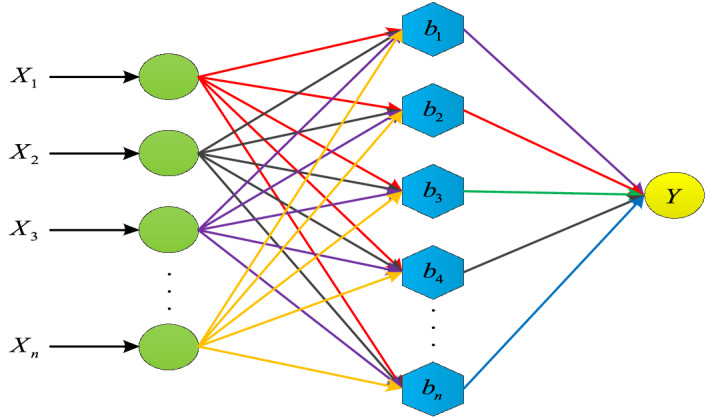
BP neural network structure.

BPNN typically have three layers: an input layer, an output layer, and a hidden layer. It takes the data into the network, analyzes each layer, and then calculates the discrepancy between the output expected and the output actually produced. This discrepancy is then used to estimate the output layer's previous layer's mistake and to update the previous layer's error (input layer). This method yields the error estimation for each layer of the neural network structure. Finally, until the best output result is attained, the network's weight and threshold are adjusted once again. The structure of BP neural network is shown in Fig. 7, and the core formula is as follows:

13 $$ Z_{k} = f_{1} \left( {\sum\limits_{i = 1}^{n} {w_{ki} x_{i} + \theta_{k} } } \right)\quad k = 1,2, \ldots ,r $$

14 $$ y_{j} = f_{2} \left( {\sum\limits_{k = 1}^{n} {v_{jk} z_{k} + \gamma_{j} } } \right)\quad j = 1,2, \ldots ,m $$

15 $$ E = \frac{1}{2}\sum\limits_{j = 1}^{m} {(t_{j} - y_{j} )^{2} = \frac{1}{2}(T - Y)^{T} (T - Y) = \frac{1}{2}e^{T} e} $$

Equation (13) is the output of the k-th node of the implied layer, where $w_{ki} x_{i} + \theta_{k}$ is the input of information $x_{i}$ at the k-th node and $Z_{k}$ is the input information of the output layer. The output of the j-th node of the output layer is like a Eq. (14), where $v_{jk} z_{k} + \gamma_{j}$ is the input of input information $Z_{k}$ at the j-th node of the output layer. The error function E of the expected output and the true output of the sample network is the Eq. (15), where $T = (t_{1} ,t_{2} ,...t_{m} )^{T}$ is the true output of the network and $e = (e_{1} ,e_{2} ,...,e_{m} )^{T}$ is the error vector.

The classification accuracy of the BPNN is influenced by the initial weight and threshold because these parameters are often created at random. A local optimal solution is easily reached because the BPNN uses the gradient descent method as the update parameter. To create the prediction model of the simultaneous integrated learning of GPSAPSO and BPNN, we decided to introduce PSO method and improve the original PSO algorithm.

### Parallel integrated learning algorithm based on GPSAPSO-BP neural network

Using GPSAPSO algorithm to optimize the weight and threshold parameters of BPNN, a parallel integrated learning algorithm based on GPSAPSO-BPNN is constructed. The idea is to determine the number of intermediate layer nodes of the network based on the input and output parameters and to obtain the number of ownership values and thresholds so as to determine the coding length of the individuals of the particle population, i.e., each individual in the population contains all the weights and thresholds. The initial weights and thresholds of the whole network are obtained by decoding the individuals. The training sample data are used to train the network, while the test sample data are used to obtain the error values. The error values are the fitness values for the GPSAPSO algorithm. The GPSAPSO algorithm is then used to optimize the weights and thresholds of the BP neural network, which results in a parallel interactive integrated learning algorithm of the two algorithms.

**Figure 8:**
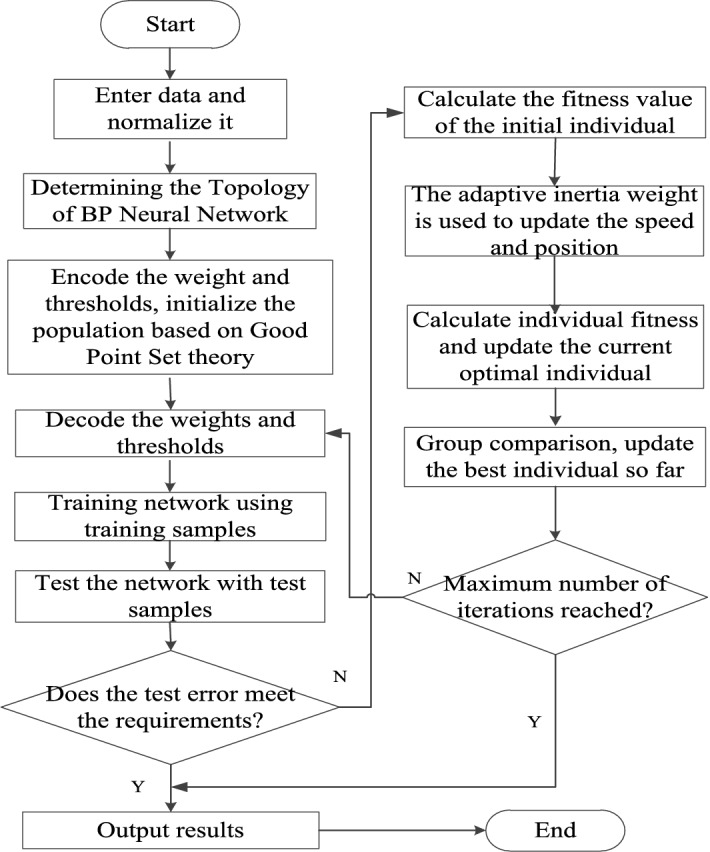
GPSAPSO-BPNN integrated algorithm flow chart.

The logical structure of GPSAPSO-BPNN algorithm is shown in Fig. 8. The specific experimental steps are as follows:

**Step 1** Input the original data (indicators and output results), and divide the test set and training set; Normalize the data;

**Step 2** Determine the number of nodes in the middle layer of BPNN, construct the structure of BPNN, initialize the weights and thresholds of BPNN, and generate individual population with uniform distribution by using good point set theory;

**Step 3** GPSAPSO initialization: Determine the maximum number of iterations $ger$, population size $popsize$, individual and social learning factors ${c}_{1}$ and ${c}_{2}$, and inertia weights $w$. The parameters are automatically adjusted with the number of iterations.

**Step 4** BPNN is optimized using GPSAPSO algorithm. Using the mean square error predicted by BP network as the optimization process, the mean square errors under different number of hidden layer nodes are compared, and the algorithm of GPSAPSO is executed through speed and position updates.

**Step 5** The BPNN optimized by GPSAPSO is trained and predicted, and the predicted value of GPSPSO-BPNN algorithm is compared with the true value.

**Step 6** Determine whether the test error meets the target requirements, and if it meets the requirements, get the best BPNN weights and thresholds, produce output results, and end the algorithm; Otherwise, judge if the GPSAPSO algorithm has reached the maximum number of iterations, and turn to Step 4, if it has not reached the maximum number of iterations. Otherwise, the network weights and thresholds will be obtained, the output results will be generated, and the algorithm will be ended.

### Data sampling and processing

The experimental data comes from the National Surface Water Quality Auto-monitoring Implementation Data Publishing System (www.cnemc.cn), which is collected by Qingyue (data.epmap.org). The experimental process chooses 12,138 data from all water quality monitoring sections of Huai River Basin in Bengbu City from January 1, 2021 to October 31, 2021.

The data are categorized for independent experiments according to various areas to ensure the accuracy and comparability of the results. Hugou, mohekou, longkang, guanzui, wuhe, bengbu zhashang, and bengbu guzhen are the names of the data Regions on the water quality monitoring is kept every four hours, the details are shown in Table 7. As the experimental object, we decide to use the data at 12:00 every day between January 1 and October31, 2021.

**Table 7:** Section classification and data volume.

Section name	Data volume	Amount of filtered data
Hu Gou	1782	260
Long Kang	1766	275
Guan Zui	1685	266
Five rivers	1791	284
Bengbu Guzhen	1780	273
Bengbu gate	1768	247
Total	12,138	1859

According to the environmental quality standard for surface water issued by the state and combined with the indicators provided by the water quality detection system, seven indicators ($X_{1}$–$X_{7}$) including pH, dissolved oxygen (mg/L), potassium permanganate index (mg/L), ammonia nitrogen (mg/L), total phosphorus (mg/L), total nitrogen (mg/L) and turbidity (NTU) are selected as the standards for water quality prediction and evaluation.

According to the standards issued by the state, the surface water quality can be divided into five grades: I, II, III, IV and V, those lower than the class V water quality standard are uniformly called inferior class V water. In order to verify the accuracy of the data, remove the monitoring data stored by the monitoring station and the date of certain lost data due to issues with monitoring station maintenance and data loss during the collection of original data; then, the data are tested and processed according to the triple standard deviation criterion to eliminate some abnormal values. The data is normalized because of the disparity in the dimensions and magnitude of the data. The water quality grade is replaced by Arabic numerals, and the inferior grade V is replaced by the number 6; the final raw data processed are shown in Tables 7 and 8 for the amount of data and partial data, independent experiments are carried out according to the section classification. Due to the limitation of space, it is only described through the section of Guzhen, Bengbu.

**Table 8:** Partial sample data.

Sample	Water quality monitoring index							Grade
	$X_{1}$	$X_{2}$	$X_{3}$	$X_{4}$	$X_{5}$	$X_{6}$	$X_{7}$	$Y$
$S_{1}$	0.6860	0.7858	0.3997	0.0566	0.0677	0.3079	0.1852	4
$S_{2}$	0.7616	0.7742	0.3388	0.0462	0.0342	0.3222	0.0949	3
$S_{3}$	0.6860	0.7329	0.3436	0.0473	0.0366	0.2709	0.1250	3
$S_{4}$	0.7442	0.7593	0.3404	0.1212	0.0748	0.2596	0.0565	3
$S_{5}$	0.5233	0.1216	0.2007	0.4836	0.3662	0.1862	0.3045	5
$S_{6}$	0.5756	0.0655	0.0558	0.2395	0.3851	0.1932	0.0000	6
$S_{7}$	0.7616	0.7329	0.3372	0.0658	0.1011	0.2880	0.2412	3
$S_{8}$	0.7500	0.7044	0.4286	0.0855	0.2398	0.3705	0.5556	4
…	…	…	…	…	…	…	…	…
$S_{n}$	0.6221	0.5732	0.0561	0.1435	0.1920	0.7289	0.2406	3

Based on the above data processing, the above seven indicators are selected as the input layer of BP network by the water quality monitoring standard, that is, the number of input layer nodes m = 7 is determined, and the corresponding output layer nodes n = 1 is determined. The number of nodes in the hidden layer is set mainly according to the empirical formula:

12 $$ h = \sqrt {m + n} + a \left( {{\text{a take an integer of 1}} - {1}0} \right) $$

By changing the value of a to test the training set, select the value with the lowest mean square error on the test set as the number of hidden layer nodes. The result of this experiment shows that the best number of hidden layer nodes is 10, and the corresponding mean square error is the lowest of 0.024789, then h = 10. Thus, a total of 80 ($m \times h + n \times h$) weights and 11 ($h + n$) thresholds are determined for the network, i.e. dim = 91 for the network parameters to be optimized (that is, the dimensions in the above algorithm). In this experiment, one-fourth of the samples are selected as the test set, while the rest of the samples are used as the training set and classified according to the section.

**Figure 9:**
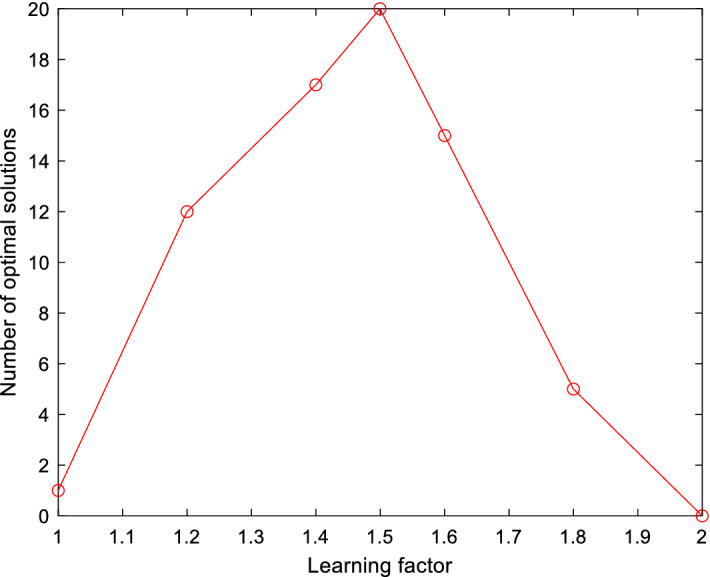
Influence of different learning factors on algorithm performance.

The learning factor of particle swarm algorithm has a great impact on the performance of the algorithm. By limiting the learning factor to [1, 2], running the algorithm 20 times while keeping other parameters unchanged, the number of times to obtain the optimal solution is taken as the evaluation criterion, and then the most appropriate learning factor is selected, as shown in Fig. 9.

Thus, the parameters of the GPSAPSO-BP algorithm are set as follows: the maximum number of evolutions $ger$ = 50, the individual learning factor $c_{1}$ = 1.4995, the social learning factor $c_{2}$ = 1.4995, and the inertia weight as above is the adaptive weight.

### Experimental results and discussion

Based on the above algorithm design and data collection and processing, the processed data are selected as the input data for the GPSAPSO-BPNN algorithm, of which 1/4 is used as the test set and the rest as the training set. The optimal threshold and weight values of the corresponding networks are obtained by running the algorithm. The fitness function is selected as the mean square error of the training and test sets, as described above.

**Figure 10:**
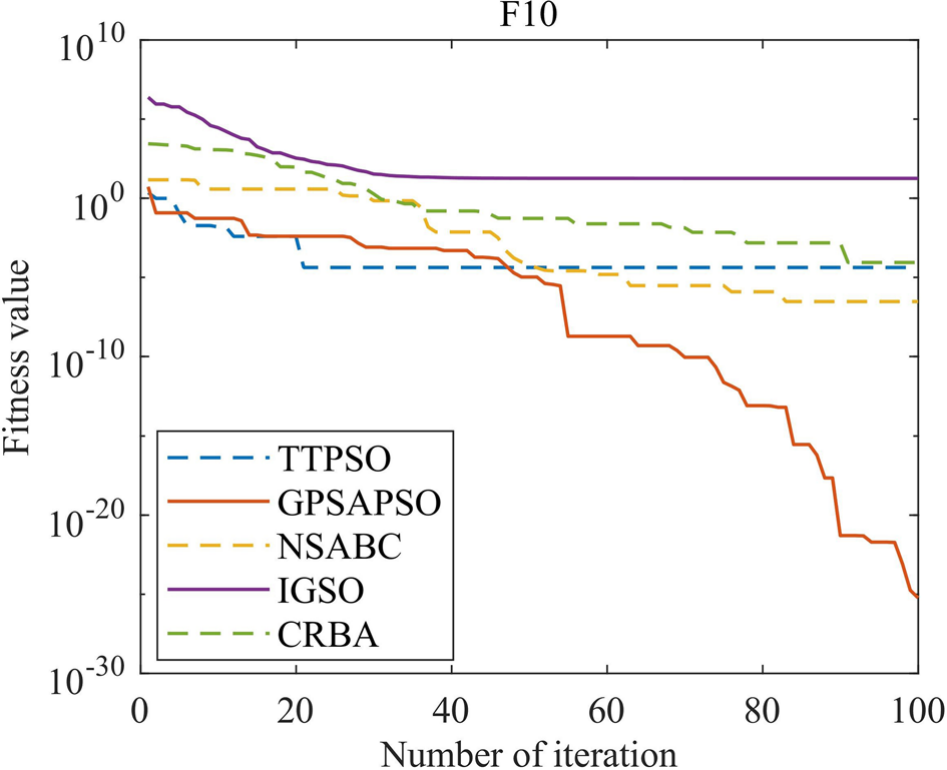
Algorithm convergence curve.

At the same time, in order to increase the reliability of the experiment, the iterations of the traditional BPNN, TTPSO-BPNN, NSABC-BPNN, IGSO-BPNN, CRBA-BPNN, and GPSAPSO-BPNN algorithms were added process and test set mean square error comparison. The same data were used for the experiments, and the training results and iteration curves are shown in Table 9 and Fig. 10.

**Table 9:** Error comparison of different algorithms.

Algorithm model	MAE	MSE	RMSE	MAPE (%)
BPNN	0.5127	0.3929	0.6268	13.1547
TTPSO-BPNN	0.1561	0.0815	0.2854	4.3648
NSABC-BPNN	0.3286	0.1967	0.4435	8.8151
IGSO-BPNN	0.2200	0.0977	0.3126	6.2861
CRBA-BPNN	0.2026	0.0788	0.2808	5.4772
GPSAPSO-BPNN	0.1447	0.0607	0.2465	3.7680

In terms of training accuracy and convergence speed, the GPSAPSO-BP algorithm significantly outperforms other algorithms based on the aforementioned experimental results. It is easy to see that for the same number of iterations, the GPSAPSO-BPNN algorithm has lower mean square error values than the BPNN, TTPSO-BPNN, NSABC-BPNN, IGSO-BPNN, and CRBA-BPNN algorithms, and higher solution speed than the other eight algorithms. The comparison of the mean absolute error, mean square error, root mean square error and error percentage of the algorithm is shown in Table 9. It can be seen that the GPSAPSO-BPNN algorithm has been improved on the basis of the original one, which proves the feasibility and advantages of the algorithm and can improve the accuracy of water quality prediction in Huai River.

## Conclusion

The theory of good point sets is introduced, an initial particle swarm with uniform distribution in the solution space is built, adaptive inertia weights are used to dynamically adjust the size of inertia weights during iteration, and finally, a good point set and an improved GPSAPSO algorithm with adaptive inertia weights are obtained through a variety of cluster co-evolution strategies. The feasibility of the GPSAPSO algorithm is verified by comparing it with TTPSO, NSABC, IGSO and CRBA through sixteen standard test functions from two to high dimensions. Finally, a parallel integrated learning model for water quality prediction of the Huaihe River was established by combining the GPSAPSO algorithm with the traditional BPNN. TTPSO-BPNN, NSABC-BPNN, IGSO-BPNN and CRBA-BPNN algorithms were added to the actual water quality prediction of Huaihe River in order to compare with GPSAPSO-BP algorithm and further verify its efficacy, including faster convergence speed and higher accuracy. Therefore, the research results of this paper provide a scientific method and basis for the monitoring and management of water quality (“Supplementary information S1”).

## Supplementary Information

Supplementary Information.

## Data Availability

The data used in the article were obtained from the China General Environmental Monitoring Station www.cnemc.cn), collected by Qingyue Data (data.epmap.org), and the water quality data recorded at 12:00 daily were selected by the authors, and the final data used were obtained by removing missing values as well as filtering outliers under the principle of triple standard deviation.

## References

[1] Su, S.S.W., & Kek, S.L. An improvement of stochastic gradient descent approach for mean-variance portfolio optimization problem. *J. Math.* (2021).

[2] Li G (2019). An improved butterfly optimization algorithm for engineering design problems using the cross-entropy method. Symmetry..

[3] Vimal V (2021). Artificial intelligence-based novel scheme for location area planning in cellular networks. Comput. Intell..

[4] Melin P, Sánchez D (2018). Multi-objective optimization for modular granular neural networks applied to pattern recognition. Inf. Sci..

[5] Holl JH (1992). Genetic algorithms. Sci. Am..

[6] Eberhart R, Kennedy J (1995). Particle swarm optimization. Proc. IEEE Inter Conf. Neural Netw..

[7] Krishnanand KN, Ghose D (2006). Glowworm swarm based optimization algorithm for multimodal functions with collective robotics applications. Multiagent Grid Syst..

[8] Karaboga, D. & Basturk, B. Artifcial bee colony (abc) optimization algorithm for solving constrained optimization problems. in *Foundations of Fuzzy Logic and Soft Computing. IFSA 2007. Lecture Notes in Computer Science*, 789–798 (Springer, 2007).

[9] Mehrabian AR, Lucas C (2006). A novel numerical optimization algorithm inspired from weed colonization. Eco. Inform..

[10] Yang, X. S., & Deb, S. Cuckoo search via Lévy flights. in *2009 World Congress on Nature & Biologically Inspired Computing (NaBIC),* 210–214 (2009).

[11] Yang XS (2010). A New Metaheuristic Bat-Inspired Algorithm.

[12] Pan WT (2012). A new fruit fly optimization algorithm: Taking the financial distress model as an example. Knowl.-Based Syst..

[13] Mirjalili S, Lewis A (2016). The whale optimization algorithm. Adv. Eng. Sofw..

[14] Mirjalili S, Mirjalili SM, Lewis A (2014). Grey wolf optimizer. Adv. Eng. Sofw..

[15] Mirjalili S (2015). The ant lion optimizer. Adv. Eng. Softw..

[16] Mirjalili S (2017). Salp Swarm Algorithm: A bio-inspired optimizer for engineering design problems. Adv. Eng. Sofw..

[17] Arora, S., S. Singh. Butterfly optimization algorithm: a novel approach for global optimization. *Soft Comput*. (2018).

[18] Heidari AA (2019). Harris hawks optimization: Algorithm and applications. Futur. Gener. Comput. Syst..

[19] Li S (2020). Slime mould algorithm: A new method for stochastic optimization. Futur. Gener. Comput. Syst..

[20] Van den Eynde J (2022). Artificial intelligence in pediatric cardiology: Taking baby steps in the big world of data. Curr. Opin. Cardiol..

[21] Guo, R., Ding, J., & Zang, W. Music online education reform and wireless network optimization using artificial intelligence piano teaching. *Wireless Commun. Mobile Comput*. (2021).

[22] Du, C., *et al*. Research on the application of artificial intelligence method in automobile engine fault diagnosis. *Eng. Res. Express*. **3**(2), (2021).

[23] Hou H, Tang K, Liu X, Zhou Y (2021). Application of artificial intelligence technology optimized by deep learning to rural financial development and rural governance. J. Glob. Inf. Manag..

[24] Abbasimehr, H., M. Shabani, & M. Yousefi. An optimized model using LSTM network for demand forecasting. *Comput. Ind. Eng*. **143,** (2020).

[25] Martínez-Santos, P., et al. Predictive mapping of aquatic ecosystems by means of support vector machines and random forests. *J. Hydrol. ***595,** (2021).

[26] Zhu S, Heddam S (2020). Prediction of dissolved oxygen in urban rivers at the Three Gorges Reservoir, China: Extreme learning machines (ELM) versus artificial neural network (ANN). Water Qual. Res. J..

[27] Shan, J., & Wang, H. Software enterprise risk detection model based on BP neural network. *Wireless Commun. Mobile Comput*. (2022).

[28] Zhai, M. Risk prediction and response strategies in corporate financial management based on optimized BP neural network. *Complexity*. (2021).

[29] Zhang, L., Gao, T., Cai, G., & Hai, K. L. Research on electric vehicle charging safety warning model based on back propagation neural network optimized by improved gray wolf algorithm. *J. Energy Storage*. **49**, (2022).

[30] Zhang WS, Hao ZQ, Zhu JJ, Du TT, Hao HM (2020). BP neural network model for short-time traffic flow forecasting based on transformed grey wolf optimizer algorithm. J. Transp. Syst. Eng. Inf. Technol..

[31] Xia, X. Study on the application of BP neural network in air quality prediction based on adaptive chaos fruit fly optimization algorithm. in *MATEC Web of Conferences,* **336**, (2021).

[32] Ebrahimi E (2016). Prediction and optimization of back-break and rock fragmentation using an artificial neural network and a bee colony algorithm. Bull. Eng. Geol. Environ..

[33] Ghosh S, Dubey AK, Das AK (2020). Numerical inspection of heterogeneity in materials using 2D heat-conduction and hybrid GA-tuned neural-network. Appl. Artif. Intell..

[34] Qian S, Wu H, Xu G (2020). An improved particle swarm optimization with clone selection principle for dynamic economic emission dispatch. Soft. Comput..

[35] Wu P, Gao L, Zou D, Li S (2011). An improved particle swarm optimization algorithm for reliability problems. ISA Trans..

[36] Dong J, Li Y, Wang M (2019). Fast multi-objective antenna optimization based on RBF neural network surrogate model optimized by improved PSO algorithm. Appl. Sci..

[37] Zhang J, Zhai Y, Han Z, Lu J (2021). Improved particle swarm optimization based on entropy and its application in implicit generalized predictive control. Entropy.

[38] Kuo, J., & Sheppard, J. W. Tournament topology particle swarm optimization. in *2021 IEEE Congress on Evolutionary Computation (CEC)*, 2265–2272 (2021).

[39] Wang H, Wang W, Xiao S, Cui Z, Xu M, Zhou X (2020). Improving artificial bee colony algorithm using a new neighborhood selection mechanism. Inf. Sci..

[40] Li D, Peng J, He D (2021). Aero-engine exhaust gas temperature prediction based on Light GBM optimized by improved bat algorithm. Therm. Sci..

[41] Li J, Li X, Dai DRS, Zhu X (2020). Research on credit risk measurement of small and micro enterprises based on the integrated algorithm of improved GSO and ELM. Math. Problems Eng..

[42] Hua LK, Wang Y (1972). Applications of Number Theory to Numerical Analysis.

[43] Wilcoxon F (1992). Breakthroughs in Statistics. Individual Comparisons by Ranking Methods.

